# Allergy to ophthalmic solutions

**DOI:** 10.1186/2045-7022-4-S3-P85

**Published:** 2014-07-18

**Authors:** Hilde Lapeere, Jolien Veramme, Julie De Zaeytijd, Jo Lambert

**Affiliations:** 1 University Hospital Ghent, Dermatology, Belgium; 2 University Hospital Ghent, Ophthalmology, Belgium

## Background

Ranibizumab (Lucentis®) is an antibody fragment with high binding affinity for all isoforms of Vascular Endothelial Growth Factor A (VEGF-A) and is specifically developed for intraocular use. Since its introduction in 2007 it has been administered increasingly and in our hospital approximately 1650 intravitreal injections were given in 2013. Ranibizumab is injected monthly and in most patients for a prolonged period of time. During the procedure several ophthalmic solutions are used, such as local anaesthetic, disinfection, mydriatic drugs and antibiotics.

## Methods

From March 2012 to January 2014, 15 patients who developed a skin reaction around the eye after intravitreal injection were tested. Patch testing and readings were performed according to the ICDRG criteria. All patients were patch tested with the Belgian standard series, cosmetic, pharmaceutical and ophthalmic series. All eyedrops used during the intravitreal injection procedure were tested. Ranibizumab could not be tested because it is expensive.

## Results

Of the 15 patients referred for patch testing 47% were females, the mean age of the patients was 70 years (range 49-87). All patients described a similar reaction pattern, i.e. a burning and stinging sensation, redness and swelling, in the majority of cases starting within 12 hours after the injection. The mean number of injections before a reaction occurred was 12. Eight patients tested positive for phenylephrine (=PE) hydrochloride 10% aqua (of which 2 also reacted to Tobrex and another 2 to Isobetadine oculaire), 2 patients reacted to sodiummetabisulfite (a preservative in Phenylephrine Minims) and 1 patient only had a positive test for Phenylephrine Minims. In 2 patients there was only one positive test for Isobetadine oculaire, in 2 patients all tests were negative.

## Conclusion

In the last two years there has been a steep rise in the number of patients with suspected contact dermatitis after injection with ranibizumab. The most common relevant allergen was PE, being positive in 60% of the patients. We hypothesize that patients become sensitized because of the high frequency with which PE is used. The rise in referrals is also due to the fact that the ophthalmologists in our hospital are aware of the problem. In literature PE has been found to be a frequent cause of contact dermatitis [1].

## References

[B1] Villareal O Reliability of diagnostic tests for contact allergy to mydriatic eyedrops Contact Dermatitis 1998 38 150 154 10.1111/j.1600-0536.1998.tb05682.x 9536407

